# Medical News

**Published:** 1853-07

**Authors:** 


### MEDICAL NEWS.

### MEDICAL GRADUATES IN 1853.

| | |
|---|---|
| Jefferson Medical College, . . . . | 223 |
| University of Pennsylvania, . . . | 160 |
| University of Maryland, . . . . . | 60 |
| Kentucky Medical School, . . . . | 39 |
| Medical Department of Yale College, . . | 15 |
| Medical Department of Pennsylvania College, . | 56 |
| Medical School of Harvard University, . | 17 |
| Starling Medical College, . . . . . | 42 |
| College of Physicians and Surgeons, N. Y., . | 53 |
| Medical College of Georgia, . . . . . | 50 |
| Medical Department of the St. Louis University, | 33 |
| Medical Department of the University of Missouri, | 26 |
| Philadelphia Medical College, . . . | 27 |
| University of Nashville, Tenn., . . . . | 36 |
| Medical Department of Hampden Sydney College, Va., | 26 |
| University of Louisville, . . . . . | 87 |

Quackery in Paris.—A case of some interest came the other day before the Correctional Tribunal. A medical man, named De Bonnard, was cited for acting illegally in administering to his patients medical preparations of a composition not set down in the codex. It appeared from the evidence, that in consequence of information given to the police, a search was made in December at the residence of Dr. de Bonnard, and a small box was seized there containing 160 little glass bottles of various medicaments, and 1350 little glass tubes filled with white globules. The object of these preparations was to enable the medical man to administer his remedy to the patient on the instant. An analysis has proved that these preparations were not in accordance with anything set down in the codex, and in consequence Dr. de Bonnard was accused of practising medicine in an illegal manner, and of selling secret remedies. The court decided that there was nothing to prove the latter count of the charge, but that there could be no doubt as to the former. Dr. de Bonnard was then sentenced to pay a fine of 100 francs.—*Dublin Med. Press*, from *Galignani's Journal.*

LIST OF DELEGATES IN ATTENDANCE, AT THE MEETING OF THE AMERICAN ASSOCIATION,

*Held in N. York, 3d, 4th and 5th May,* 1853.

* Killed by the catastrophe at Norwalk.

*Maine.*—John Benson, Chas. S. D. Fessenden, Alonzo Garcelon, Isaac Lincoln, James McKean, E. R. Peaslee, T. G. Stockbridge.

*New Hampshire.*—*Josiah Bartlett, Dixi Crosby, Josiah Crosby, G. W. Garland, Luther M. Knight, Alvah Moulton, Edw. H. Parker, E. R. Peaslee, Nath. Sanborne, A. Smalley, Albert Smith, W. P. Stone, E. K Webster.

*Vermont.*—Chas. L. Allen, H. B. Brown, E. S. Carr, C. H. Cleveland, Earl Cushman, C. L. Ford, Selim Newell, A. T. Woodward.

*Massachusetts.*—Nathan Adams, Z. B. Adams, Ebenezer Alden, Nathan Allen, John B. Alley, George Bartlett, Lyman Bartlett, Jonas W. Bemis, Henry G. Bigelow, J. Nelson Borland, Thos. R. Boutelle, H. I. Bowditch, Jonathan Brown, Charles H. Browne, S. G. Burnap, Ephraim Buck, E W. Carpenter, Thos. L. Chapman, George Choate, Henry G. Clark, Moses Clark, Daniel T. Coit, Clarkson T. Collins, Charles A. Davis, Lemuel Dickerman, Hanover Dickey, Wm. Dickinson, Calvin Ellis, J. Farnum, Samuel A. Fisk, Calvin T. Fiske, Edward Flint, Geo. H. Gay, Augustus A. Gould, *James H. Gray, John Green, Robert Greer, Benj. F. Heywood, Alfred Hitchcock, C. C. Holmes, O. W. Holmes, Charles D. Homans, R. W. Hooper, Daniel Hovey, Charles F. Jackson, John Jeffries, J. P. Jewett, N. C. Keep, Gilman Kimball, Wm. D. Lamb, Erasmus D. Miller, Josiah Noyes, James M. Nye, J. W. D. Osgood, Thaddeus Phelps, *A. L. Peirson, Edward Reynolds, Samuel Richardson, Ira Sampson, Augustine Shurtleff, Benj. F. Smith, *James M. Smith, Seth L. Sprague, Henry O. Stone, D. H. Storer, Daniel Thompson, S. D. Townsend, E. G. Tucker, Charles E. Ware, J. C. Warren, J. M. Warren, Moses W. Weld, Wm. G. Wheeler, Vassel White, Wm. C. Whitmilge, Adams Wiley, H. W. Williams, Edward Worcester, Wm. Workman.

*Rhode Island.*—Hiram Allen, S. Augustus Arnold, Richmond Brownell, George Capron, Sylvanus Clapp, Hiram Cleveland, Theophilus C. Dunn, Ezekiel Fowler, David King, Lewis L. Miller, I. Mauran, Charles W. Parsons, Usher Parsons, Henry W. Rivers, Edwin M. Snow.

*Connecticut.*—Rufus Baker, A. W. Barrows, *Samuel Beach, Sheldon Beardsley, L. N. Beardsley, H. N. Bennett, S. C. Beresford, E. H. Bishop, G. G. Bissel, D. E. Bostwick, Henry Bronson, Clarence M. Brownell, Wm. B. Casey, B. H. Caslin, Andrew Castle, Samuel Catlin, Jr., F. L. Dickinson, Johnson C. Hatch, Roswell Hawley, Charles Hooker,

Worthington Hooker, Robt. Hubbard, Samuel Hutchings, A. M. Huxby, Nathan B. Ives, P. A. Jewett, Jonathan Knight, Elijah Middlebrook, A. Moody, Nathan P. Pike, Gurdon W. Russel, F. W. Shepard, Alvan Talcott, *Archibald Welch, Benjamin Welch, R. A. White, Datus Williams, Orson Wood, William Wood, Ashbel Woodward, S. Woodruff.

*New York.*—John G. Adams, Lucius H. Allen, James Anderson, James H. Armsby, John H. Arnold, David F. Atwater, James M. Austin, Daniel Ayres, M. N. Babcock, John Ball, B. Fordyce Barker, J. P. Batchelder, William Bay, E. L. Beadle, Wm. H. Beardsley, Seneca Beebe, A. G. Bigelow, Wm. N. Blakeman, Thos. W. Blatchford, James C. Bliss, S. V. R. Bogert, Jackson Bolton, Reed B. Bontecou, J. H. Borrowe, Sumner Ely, John T. Ferguson, Austin Flint, C. L. Ford, Joel Foster, S. Consant Foster, John W. Francis, A. K. Gardner, J. P. Garrish, Anthony Gescheidt, C. R. Gilman, Caleb Green, Horace Green, Isaac Greene, John H. Griscom, F. H. Hamilton, Elisha Harris, John Hart, Charles Henschell, James Hibben, Richard K. Hoffman, S. T. Hubbard, Frederick Hyde, Ferris Jacobs, Charles G. Pomeroy, James O. Pond, Alfred C. Post, J. Purdy, S. A. Purdy, S. S. Purple, C. H. Raymond, D. M. Reese, J. H. Reynolds, T. B. Reynolds, Wm. Rockwell, L. A. Sayre, A. L. Saunders, Earnest Schilling, Albert Smith, Charles D. Smith, Joseph M. Smith, Horatio S. Smith, Stephen Smith, Simeon Snow, Thomas Spencer, J. S. Sprague, B. P. Staats, F. Campbell Stewart, Thomas C. Brinsmade, P. B. Brooks, Gurdon Buck, Wm. P. Buel, Henry D. Bulkley, Wm. C. Butler, George N. Burwell, G. P. Camman, J. M. Carnochan, Edson Carr, Galen Carter, Jonathan H. Case, M. H. Cash, John C. Cheesman, H. S. Chubbuck, Wm. Henry Church, J. W. G. Clements, Thomas Cock, Thomas F. Cock, James Cockroft, A. B. Coe, James S. Cooper, John W. Corson, C. B. Coventry, Henry G. Cox, Richard O. Crandall, John C. Dalton, Jr., E. H. Davis, Edward Delafield, William Detmold, D. B. Devendorf, A. F. Doolittle, Henry S. Downs, Abram Dubois, *Wm. C. Dwight, Pliny Earle, H. N. Eastman, Wm. H. Jackson, J. Foster Jenkins, Harvey Jewett, F. U. Johnston, David T. Jones, Stephen S. Keene, R. S. Kissam, Jonathan S. Lawrence, B. C. Leveridge, Jared Linsly, John McCall, B. W. McCready, Ebenezer McFarlan, W. H. Macneven, James McNaughton, Leonard C. McPhail, Alden March, T. M. Markoe, Wm. H. Maxwell, John T. Metcalfe, John Miller, S. R. Millington, J. M. Minor, Henry Mitchell, R. C. Morris, Valentine Mott, Walter Mott, Peter Moulton, E. S. Nichols, Eugene O'Donoughue, Benjamin Ogden, Samuel J. Osborn, Andrew Otterson, Willard Parker, George A. Peters, James L. Phelps, S. B. Phillips, James Stewart, Philander Stewart, Alex. H. Stevens, Mark Stephenson, John O. Stone, John A. Swett, John Swinburne, Isaac E. Taylor, William Taylor, Alex. Thompson, A. G. Thompson, John Trenor, Henry Van Arsdale, Wm. H. Van Buren, Peter Van Buren, Sam. O. Vanderpoel, J. R. Van Fleck, M. D. Van Pelt, James Warren, John Watson, Robert Watts, Cyrus Weeks, H. S. West, Lewis C. Wheeler, James P. White, Devillo White, Oliver White, S. P. White, Charles H. Wilcox, Augustus Willard, Nelson

Winton, Isaac Wood, James R. Wood, Geo. F. Woodward, Joseph Worster.

*New Jersey.*—B. Rush Bateman, P. T. Brakely, A. E. Budd, Charles Butcher, S. W. Butler, J. W. Canfield, G. R. Chetwood, Henry J. Clark, L. Condict, Charles Cook, J. M. Cornelison, J. W. Craig, Alex. N. Dougherty, Franklin Gauntt, John Grimes, John B. Johnes, Samuel Lily, A. Linn, S. R. Millington, A. D. Morford, J. B. Munn, Sam'l H. Pennington, W. Pierson, John H. Phillips, Alex. W. Rogers, L. A. Smith, Othniel H. Taylor, Theodore R. Varick, A. F. Voorhies, John F. Ward.

*Pennsylvania.*—J. M. Allen, William Ashmead, John L. Atlee, Thomas F. Betton, C. H. Bibighaus, John B. Biddle, B. Bohren, Henry Bond, John B. Brinton, James Bryan, F. S. Burrows, Henry Carpenter, James S. Carpenter, J. Carson, P. Cassidy, Benj. H. Coates, D. F. Condie, Hiram Corson, M. R. Cryder, J. Augustus Ehler, Thomas Ellmaker, G. Emerson, Joseph P. Gazzam, Alex. K. Gaston, Traill Green, Edward Hartshorne, Isaac Hayes, Anthony Heger, Frank M. Heister, Charles Innes, Wilson Jewell, (Prof.) Samuel Jackson, Wm. V. Keating, William Keith, Alfred S. Kennedy, James W. Kerr, J. F. Lamb, R. La Roche, E. F. Leake, R. J. Levis, John Lowman, J. K. Mason, William Maybury, Charles D. Meigs, John K. Mitchell, S. N. Ogier, Joseph Pancoast, C. W. Parrish, Wm. A. Piper, John Ream, C. O. Richards, R. E. Rogers, W. S. W. Ruschenberger, John J. Reese, Francis R. Shunk, Francis Gurney Smith, Henry H. Smith, Robert K. Smith, Thomas Spencer, Alfred Stille, Moreton Stille, J. B. Stubbs, Isaac Thomas, R. H. Townsend, W. A. Van Buskirk, Isaac R. Walker, Francis West, G. W. Winley, Caspar Wister, Thomas H. Yardley, J. L. Ziegler, George J. Ziegler,

*Delaware.*—H. F. Askew, James Couper, J. F. Wilson.

*Maryland.*—Thomas E. Bond, Jr., C. C. Cox, Francis Donaldson, E. P. Duval, Washington Duval, John R. W. Dunbar, Henry T. Goldsborough, E. M. Hardcastle, F. E. B. Hentze, Joel Hopkins, P. S. Kinnernon, G. W. Miltenberger, James Wynne, Robert Murray, D. A. O'Donnell, Wm. Riley, Joseph Roby, J. E. Snodgrass, Samuel Tyler.

*District of Columbia.*—S. C. Bersey, G. M. Dove, W. L. C. Du Hamel, Samuel W. Everett, Alex. T. B. Garnett, Harvey Lindsly, A. J. Semmes, John Fred. May, Thomas Miller, James E. Morgan.

*Virginia.*—Thomas P. Atkinson, James Bolton, John Dove, P. Claiborne Gooch, Theodore Goodloe, Carter P. Johnson, Charles R. Kemper, George A. Otis, C. R. Palmore, Wm. W. Parker, Alban S. Payne, J. F. Peebles, F. W. Rodney, John F. Sinton, P. Trent, J. Wistar Walke, Beverly R. Wellford.

*North Carolina.*—J. Graham Tull, N. J. Pittman,

*South Carolina.*—R. S. Bailey, B. W. Bradley, D. J. Cain, Dan. G. Crane, Joseph S. Crane, Henry R. Frost, Peter C. Gaillard, R. A. Kinloch, Robert Lebby, John May, Middleton Michael, James Moultrie, T. L. Ogler, Thomas G. Prioleau, Wm. T. Wragg.

*Georgia.*—Wm. Gaston Bullock, Henry F. Campbell, Thomas Hoey, G. M. Kollock.

*Alabama.*—J. A. English, Thomas J. Howell, G. M. Meriwether, Robinson Miller.

*Louisiana.*—J. Hancock Douglas.

*Tennessee.*—Zeno Harris, J. B. Lindsley, Charles K. Winston.

*Kentucky.*—T. S. Bell, C. J. Blackburn, Joshua B. Flint, D. L. Freeman, T. J. Moore, B. Silliman, Jr., L. P. Yandell.

*Ohio.*—J. D. Cotton, Jacob J. Delamater, Thos. W. Gordon, R. L. Howard, J. P. Judkins, John A. Murphy, R. D. Mussey, C. H. Raymond, Wolcott Richards, S. Hanbury Smith, N. E. Soule, D. Tilden.

*Indiana.*—Geo. L. Andrews, Wm. A. Clapp, Vierlin Kersey, Wm. Lomax, Geo. M. Maclean, Hugh Ronalds, C. Landon Rose, Joseph Somes, R. R. Town.

*Illinois.*—Daniel Brainard, N. S. Davis, Joseph C. Frye, Adams Nichols, A. B. Palmer, Rudolphus Rouse, H. Williams.

*Missouri.*—C. W. Hempstead, J. B. Johnson, E. S. Lemoine, C. A. Pope, Jas. R. Washington.

*Michigan.*—Josiah Andrews, F. K. Bailey, Aaron L. Leland, Isaac Paddock, T. Pitcher, Henry Taylor.

*Iowa.*—J. C. Hughes.

*Wisconsin.*—C. B. Chapman, H. Van Dusen.

*Arkansas.*—— Boreland,

*California.*—R. Stephen Harris.

*Florida.*—Thos. King Leonard, Geo. W. Betton.

*U. S. Army.*—R. H. Coolidge, J. M. Cuyler, Josiah Simpson.

*U. S. Navy.*—B. F. Bache, Ninian Pinkney.

*Foreign.*—Robt. R. Kelley (from Beyroot, Syria.)

R. M. McIlvaine and N. J. Pittman (from Medical Society of Paris.)

